# Development of AMSTAR: a measurement tool to assess the methodological quality of systematic reviews

**DOI:** 10.1186/1471-2288-7-10

**Published:** 2007-02-15

**Authors:** Beverley J Shea, Jeremy M Grimshaw, George A Wells, Maarten Boers, Neil Andersson, Candyce Hamel, Ashley C Porter, Peter Tugwell, David Moher, Lex M Bouter

**Affiliations:** 1 EMGO Institute, VU University Medical Center, Amsterdam, the Netherlands; 2 Institute of Population Health, Ottawa, Ontario, Canada; 3 Department of Epidemiology and Community Medicine, University of Ottawa, Ottawa, Ontario, Canada; 4 Department of Clinical Epidemiology and Biostatistics. VU University Medical Center, Amsterdam, the Netherlands; 5 Community Information and Epidemiological Technologies (CIETcanada), Ottawa, Ontario, Canada; 6 University of Ottawa, Ottawa, Ontario, Canada

## Abstract

**Background:**

Our objective was to develop an instrument to assess the methodological quality of systematic reviews, building upon previous tools, empirical evidence and expert consensus.

**Methods:**

A 37-item assessment tool was formed by combining 1) the enhanced Overview Quality Assessment Questionnaire (OQAQ), 2) a checklist created by Sacks, and 3) three additional items recently judged to be of methodological importance. This tool was applied to 99 paper-based and 52 electronic systematic reviews. Exploratory factor analysis was used to identify underlying components. The results were considered by methodological experts using a nominal group technique aimed at item reduction and design of an assessment tool with face and content validity.

**Results:**

The factor analysis identified 11 components. From each component, one item was selected by the nominal group. The resulting instrument was judged to have face and content validity.

**Conclusion:**

A measurement tool for the 'assessment of multiple systematic reviews' (AMSTAR) was developed. The tool consists of 11 items and has good face and content validity for measuring the methodological quality of systematic reviews. Additional studies are needed with a focus on the reproducibility and construct validity of AMSTAR, before strong recommendations can be made on its use.

## Background

It has been estimated that healthcare professionals attempting to keep abreast of their field would need to read an average of 17–20 original articles every day [1]. Increasingly, systematic reviews are being advocated as a way to keep up with current medical literature [2]. A well conducted systematic review addresses a carefully formulated question by analyzing all available evidence. It employs an objective search of the literature,  applying predetermined inclusion and exclusion criteria to the literature, critically appraising what is found to be relevant. It then extracts and synthesizes data from the available evidence base to formulate findings [3].

However, in spite of the care with which they are conducted, systematic reviews may differ in quality, and yield different answers to the same question [4]. As a result, users of systematic reviews should be critical and look carefully at the methodological quality of the available reviews [5].

A decade has passed since the initial development of tools to assess the quality of systematic reviews, such as those created by Oxman and Guyatt [6] and Sacks [7]. There are now more than 24 instruments available to assess the quality of systematic reviews [8]. Nevertheless, the majority of the available instruments are not widely used. Several are lengthy and include complicated instructions for their use. Furthermore, since their development, considerable empirical research has accumulated about potential sources of bias in systematic reviews. For example, recent methodological research has highlighted the potential importance of publication language and publication bias in systematic reviews [9-11].

Therefore, our goal was to develop a new instrument for assessing the methodological quality of systematic reviews by building upon empirical data collected with previously developed tools and utilizing expert opinion.

This goal was pursued by two study objectives. Our first objective was to assess a large sample of systematic reviews using an item pool drawn from two available instruments used to assess methodological quality, supplemented by additional items judged to be needed on the basis of recent publications. We used exploratory factor analysis to identify the underlying component structure. Our second objective was to build on the results of this factor analysis, by using experts in a nominal group technique (NGT) to reduce the items pool and to decide on a new assessment tool with face and content validity.

## Methods

We designed a 37-item assessment tool that we developed by combining items from two available instruments: the enhanced Overview Quality Assessment Questionnaire (OQAQ) [8] containing 10 items and a checklist created by Sacks [7] containing 24 items. We supplemented this with three additional items based upon methodological advances in the field since the development of the original two instruments: *Language restriction*: Language restriction in systematic reviews remains controversial. Some studies have suggested that systematic reviews that include only English language publications tend to overestimate effect sizes [10], whereas other studies suggest that such language restrictions may not do so [11]. An item was added to determine whether a language restriction was applied in selecting studies for the systematic review. *2) Publication bias*: Publication bias refers to the tendency for research with negative findings to get published less frequently, less prominently, or more slowly, and the tendency for research with positive findings to get published more than once. Publication bias has been identified as a major threat to the validity of systematic reviews. Empirical research suggests that publication bias is widespread, and that a variety of methods are now available to assess publication bias [12-19]. An item was added to determine whether the authors assessed the likelihood of publication bias. *3) Publication status *of studies suggests that published trials are generally larger and may show an overall greater treatment effect than studies published in the 'grey' literature [20]. The importance of including grey literature in all systematic reviews has been discussed [21]. The assessment of the inclusion of grey literature considers whether or not the authors reported searching for grey literature.

### Objective 1

The 37-item assessment tool was used to appraise 99 paper-based reviews identified from a database of reviews and meta-analyses [22] and 52 Cochrane systematic reviews from the Cochrane Database of Systematic Reviews [9]. After the list of selected systematic reviews was generated, full copies of these were retrieved, copied, and masked to conceal author, institution, and journal. Reviews in languages other than English (i.e., French, German, and Portuguese) were translated into English with the assistance of colleagues before masking [23].

For each included systematic review, two reviewers independently assessed the methodological quality with the 37 items (CH, BS).

Statistical analyses and graphs displaying the results obtained were produced using SPSS version 13.0 for Windows. The 37 items were subjected to principal components analysis, and Varimax rotations were used to rotate the components. Items with low factor loadings of < 0.50 were removed.

### Objective 2

We convened an international panel of eleven experts in the fields of methodological quality assessment and systematic reviews. The group was selected from three organizations involved both in the conduct of systematic reviews and in the assessment of methodological quality. The group was made up of clinicians, methodologists and epidemiologists, and reviewers who were new to the field. Some individuals were previously involved in the Cochrane Collaboration, while a number were not. By examining the results of the factor analysis, they reflected critically on the components identified and decided on the items to be included in the new instrument. The nominal group process took place in San Francisco during a one day session.

We conducted the following NGT in order to achieve agreement. After delivery of an overview of the project and the planned process for the day, the panel reviewed the results of the factor analysis. The aim of the NGT was to structure interaction within the group. Firstly, each participant was asked to record his or her ideas independently and privately. The ideas were then listed  in a round-robin format. One idea was collected from each individual in turn and listed in front of the group by the facilitator, and the process was continued until all ideas had been listed. Individuals then privately  recorded their judgements. Subsequent discussions took place. The individual judgements were aggregated statistically to derive the group judgements. The nominal group was also asked to agree on a final label for each of the 11 components. A description was formulated for each of the items and a next-to-final instrument was assembled. This was circulated electronically to the group for a final round of fine tuning.

## Results

### Objective 1

The items were subjected to factor analysis, and only those items that loaded highly on one component (>.50) were retained. The described factor analysis made it possible to reduce the 37-item instrument to a shorter (29-item) instrument that measured 11 components (Table 1).

**Table 1:** Items identified through the factor analysis

	Original instrument (item no)	1	2	3	4	5	6	7	8	9	10	11
1 Protocol	Sacks	.58										
2 Literature Search	Sacks				.82							
3 List of Trials Analyzed	Sacks		.75									
4 Log of Rejected Trials	Sacks								.68			
5 Treatment Assignment	Sacks		.80									
6 Ranges of Patients	Sacks											
7 Range of Treatment	Sacks		.88									
8 Range of Diagnosis	Sacks		.80									
9 CombinabilityCriteria	Sacks									.88		
10 Measurement	Sacks				.57							
11 Selection Bias	Sacks	.85										
12 Data abstraction	Sacks	.50										
13 Inter-observer Agreement	Sacks	.65										
14 Sources of Support	Sacks								.64			
15 Statistical Methods	Sacks			.81								
16. Statistical Errors	Sacks											
17 Confidence Intervals	Sacks			.73								
18 Subgroup Analysis	Sacks											
19 Quality Assessment	Sacks					.77						
20 Varying Methods	Sacks					.63						
21 Publication Bias 1	Sacks						.77					
22 Caveats	Sacks											
23 Economic Impact	Sacks											.84
24 Language 1	Added to Sacks							.79				
25 Search Strategy	OQAQ (1)				.81							
26 Was the search comprehensive	OQAQ (2)											
27 Criteria used for deciding which studies to include	OQAQ (3)											
28 Was bias in the selection avoided	OQAQ (4)	.81										
29 Were the criteria used for assessing the validity reported?	OQAQ (5)					.75						
30 Was the validity of all studies referred to in the text assessed using appropriate criteria	OQAQ (6)				.53						.60	
31 Were the methods used to combine the finding of the relevant studies reported	OQAQ (7)											
32 Were the findings of the relevant studies combined appropriately	OQAQ (8)			.78								
33 Were the conclusions made by the author supported by the data	OQAQ (9)			.68								
34 Overall Summary	OQAQ (10)											
35. Publication Bias 2	Additional (1)						.80					
36 Publication Status	Additional (2)							.77				
37 Language 2	Additional (3)							.63				

### Objective 2

The nominal group discussed all 11 components (Table 1). The items most appropriate for the components (Table 2), were included in the draft instrument [also see Additional file 1]. The instrument is an 11-item questionnaire that asks reviewers to answer yes, no, can't answer or not applicable. A separate question on language was identified in the factor analysis as a significant issue, but the nominal group felt that the contradictory evidence in the literature warranted removing this item from the shortened item list and capturing it under the question on publication status.

**Table 2:** AMSTAR is a measurement tool created to assess the methodological quality of systematic reviews.

**1. Was an 'a priori' design provided?** The research question and inclusion criteria should be established before the conduct of the review.	□ Yes □ No □ Can't answer □ Not applicable
**2. Was there duplicate study selection and data extraction?** There should be at least two independent data extractors and a consensus procedure for disagreements should be in place.	□ Yes □ No □ Can't answer □ Not applicable

**3. Was a comprehensive literature search performed?** At least two electronic sources should be searched. The report must include years and databases used (e.g. Central, EMBASE, and MEDLINE). Key words and/or MESH terms must be stated and where feasible the search strategy should be provided. All searches should be supplemented by consulting current contents, reviews, textbooks, specialized registers, or experts in the particular field of study, and by reviewing the references in the studies found.	□ Yes □ No □ Can't answer □ Not applicable

**4. Was the status of publication (i.e. grey literature) used as an inclusion criterion?** The authors should state that they searched for reports regardless of their publication type. The authors should state whether or not they excluded any reports (from the systematic review), based on their publication status, language etc.	□ Yes □ No □ Can't answer □ Not applicable

**5. Was a list of studies (included and excluded) provided?** A list of included and excluded studies should be provided.	□ Yes □ No □ Can't answer □ Not applicable

**6. Were the characteristics of the included studies provided?** In an aggregated form such as a table, data from the original studies should be provided on the participants, interventions and outcomes. The ranges of characteristics in all the studies analyzed e.g. age, race, sex, relevant socioeconomic data, disease status, duration, severity, or other diseases should be reported.	□ Yes □ No □ Can't answer □ Not applicable

**7. Was the scientific quality of the included studies assessed and documented?** 'A priori' methods of assessment should be provided (e.g., for effectiveness studies if the author(s) chose to include only randomized, double-blind, placebo controlled studies, or allocation concealment as inclusion criteria); for other types of studies alternative items will be relevant.	□ Yes □ No □ Can't answer □ Not applicable

**8. Was the scientific quality of the included studies used appropriately in formulating conclusions?** The results of the methodological rigor and scientific quality should be considered in the analysis and the conclusions of the review, and explicitly stated in formulating recommendations.	□ Yes □ No □ Can't answer □ Not applicable

**9. Were the methods used to combine the findings of studies appropriate?** For the pooled results, a test should be done to ensure the studies were combinable, to assess their homogeneity (i.e. Chi-squared test for homogeneity, I^2^). If heterogeneity exists a random effects model should be used and/or the clinical appropriateness of combining should be taken into consideration (i.e. is it sensible to combine?).	□ Yes □ No □ Can't answer □ Not applicable

**10. Was the likelihood of publication bias assessed?** An assessment of publication bias should include a combination of graphical aids (e.g., funnel plot, other available tests) and/or statistical tests (e.g., Egger regression test).	□ Yes □ No □ Can't answer □ Not applicable

**11. Was the conflict of interest stated?** Potential sources of support should be clearly acknowledged in both the systematic review and the included studies.	□ Yes □ No □ Can't answer □ Not applicable

## Discussion

### Strengths and Weaknesses

Our purpose was to help users of systematic reviews to critically appraise systematic reviews. Therefore, we set out with the goal of developing a new instrument for assessing the methodological quality of systematic reviews, by building upon empirical data on previously developed tools, empirical evidence and utilizing expert opinion.

Because we had already created a dataset of 151 systematic reviews assessed using 37 completed items for each review, we were able to conduct a factor analysis as the first step in the creation of the new tool. A more commonly used approach would have been to harvest appropriate items from existing questionnaires. This method has been used extensively in the development of instruments for assessing the quality of both randomized and non-randomized studies of health care interventions [24-26]. The disadvantage of harvesting appropriate items from existing questionnaires is that it relies heavily on the validation of the source questionnaires [27]. Conducting a factor analysis made it possible to determine whether the measured dimensions could in principle be assessed using a smaller number of items.

Traditionally, factor analysis is divided into two types of analyses: exploratory and confirmatory. As its name indicates, exploratory factor analysis aims to discover the main constructs or dimensions of a concept by conducting a preliminary investigation of the correlations between all the identified variables. This process is also known as Principal Components Analysis (PCA). PCA has been recommended for use in test construction by Kline, as a means of condensing the correlation matrix, rather than as an aid to the interpretation of the factor-structure of a questionnaire [28]. Items with low factor loadings tend to be weakly correlated with other items, and therefore were removed. Various rotational strategies have also been proposed. The goal of all of them is to obtain a clear pattern of loadings, that is, factors that are somehow clearly marked by high loadings for some variables and low loadings for others [29,30]. We used this approach because it is useful when a body of theory or principles has been established, but has not yet been operationalised into an evaluative framework [31].

The structured-discussion format employed in this project enabled all participants to contribute to the refining of the assessment tool. The nominal technique followed involved experts, discussion, and a consensus that was qualitative in nature. Consequently, it complemented the quantitative nature of factor analysis, and as a result the final tool had face and content validity as judged by the nominal consensus panel.

We recognize the need for further testing of AMSTAR. Additional studies are necessary with a focus on the reproducibility and construct validity of AMSTAR before strong recommendations can be made on its use. The Canadian Agency for Drugs and Technologies in Health (CADTH) [32] undertook an independent assessment of available quality assessment criteria for systematic reviews. Feedback from CADTH reviewers has been very positive. Further preliminary experience suggests that AMSTAR has good reliability and convergent validity also suggesting that appraisers can apply it in a consistent way.

AMSTAR, if used widely after external validation, could also enable methodological research (i.e. meta-regression of item of AMSTAR and effect size of reviews). Our instrument is an attempt to achieve consensus amongst current mainstream opinions. Inevitably, new evidence will modify current thinking in some areas and at that point the AMSTAR will be updated. This is indeed likely to be the case with techniques to identify and quantify publication bias [33]. Although a number of alternative tests for publication bias exist, none has yet been validated [34].

Publication bias remains an area of contention amongst those who assess the quality of systematic reviews. It remains a research priority because it is unclear what the impact of publication bias is on making decisions in health care. We are aware of the 20 years of work that has gone in this area of research. This has given us some clear answers as to the effect publication bias may have on the overall results of estimating the impact of interventions.

AMSTAR will remain a living document and advances in empirical methodological research will be reflected in further improvements to the instrument.

## Conclusion

A measurement tool for *assessment of multiple systematic reviews *(AMSTAR) was developed. The tool consists of 11 items and has good face and content validity for measuring the methodological quality of systematic reviews. Additional studies are needed with a focus on the reproducibility and construct validity of AMSTAR, before strong recommendations can be made on its use.

## Competing interests

The author(s) declare that they have no competing interests.

## Authors' contributions

BS designed and conducted the study and wrote the manuscript. MB, JG and LB participated in the design and coordination and assisted with writing the manuscript. CH and AP carried out the quality assessments and assisted with the writing. NA assisted in the conduct of the study and commented on earlier drafts. GW assisted in the design of the study and commented on the statistical analysis. PT helped with the design of the study and assisted with the nominal group process. All authors read and approved the final manuscript.

## Pre-publication history

The pre-publication history for this paper can be accessed here:



## Supplementary Material

Additional File 1 AMSTAR. AMSTAR is a measurement tool created to assess the methodological quality of systematic reviews. Click here for file
